# CANNABIS USE AND THE DEVELOPING BRAIN: HIGHS AND LOWS

**DOI:** 10.3389/frym.2023.898445

**Published:** 2023-08-16

**Authors:** Yasmin L. Hurd, Jacqueline-Marie N. Ferland, Yoko Nomura, Leslie A. Hulvershorn, Kevin M. Gray, Christian Thurstone

**Affiliations:** 1 Departments of Neuroscience and Psychiatry, Addiction Institute of Mount Sinai, Icahn School of Medicine at Mount Sinai, New York, NY, United States; 2 Department of Psychiatry, Icahn School of Medicine at Mount Sinai, New York, NY, United States; 3 Department of Environmental Medicine and Public Health, Icahn School of Medicine at Mount Sinai, New York, NY, United States; 4 Department of Psychology, Queens College and Graduate Center, City University of New York, New York, NY, United States; 5 Department of Psychiatry, Indiana University School of Medicine, Indianapolis, IN, United States; 6 Department of Psychiatry and Behavioral Sciences, Medical University of South Carolina, Charleston, SC, United States; 7 Behavioral Health—Adolescent Outpatient, Denver Health and Hospital Authority and University of Colorado School of Medicine, Denver, CO, United States

## Abstract

Although cannabis is a naturally occurring plant with a long history of use by humans, the chemicals it contains, called cannabinoids, can act on the human body in many ways. Use of cannabis during important periods of development, such as during pregnancy and adolescence, can have a long-lasting impact on the way the brain forms and develops its systems to control emotions and other functions. This article gives an overview of some of the effects of cannabinoids on the developing brain, before birth and as teenagers, and provides information about how young people can prevent or minimize the negative effects of cannabis on their brains.

## Graphical Abstract

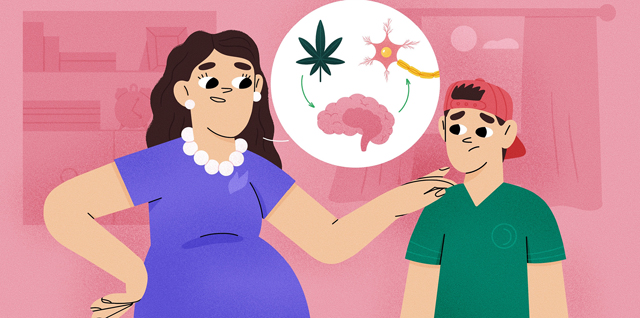

## CANNABIS AFFECTS THE BRAIN—ESPECIALLY DURING DEVELOPMENT

Cannabis is a plant also known as weed, pot, grass, bud, dank, ganja, and flower. Cannabis use in America is increasing—more teenagers now use cannabis than smoke cigarettes, although cannabis remains illegal in many countries [1]. Cannabis contains more than 500 chemicals, at least 140 of which are called cannabinoids, the active ingredients of cannabis that are responsible for the way it affects people. The main cannabinoid that causes the “high” people experience from smoking or ingesting cannabis is called delta-9-tetrahydrocannabinol, commonly known as THC.

In our bodies, we have naturally occurring cannabinoids called endocannabinoids. Endocannabinoids influence the brain and body in a very controlled way. They play a role in many normal bodily processes, including emotion control, movement, memory, and the immune system, just to name a few [2]. In the developing brain, endocannabinoids help to establish healthy connections between nerve cells, so that these cells can communicate properly. Endocannabinoids do all these things by binding to specific molecules called cannabinoid receptors, which exist on the surfaces of many cells. When THC enters the body through cannabis use, it binds to cannabinoid receptors more strongly than our own internal endocannabinoids do, and it overwhelms the body’s natural cannabinoid system.

The potency (strength) of cannabis has been increasing over the years. Before 2000, the THC concentration in street cannabis was 4% or less. Since then, however, THC levels in cannabis have consistently doubled in concentration: from 4–8% in the early 2000s to 20% now. Highly potent products such as dabs or vape pens boost THC content even more—to 70% and higher.

Given the many effects cannabis can have on the body and brain and the increased potency of cannabis available to young people, understanding how cannabis use might impact the developing brain is extremely important. Two key stages of brain development are particularly critical in terms of cannabis exposure: the prenatal (before birth) period within the womb, and the teenage years. Exposure to cannabis at either of these critical periods can increase the risk of anxiety, depression, and addiction. So, how exactly do cannabis and THC affect the developing brain?

## THC EXPOSURE IN THE WOMB

Approximately 18% of pregnant women use cannabis during pregnancy and/or while breastfeeding, which exposes their unborn babies and newborns to THC. It is known that THC binds to cannabinoid receptors in the developing brain, and studies show that babies exposed to cannabis during pregnancy are more likely to be anxious, have depression, or to be more aggressive and impulsive in childhood and adolescence [3]. Other studies show that exposure to THC in the womb changes genes related to brain cell structure and the production of certain critical proteins that regulate behavior. For example, prenatal THC exposure can reduce the number of dopamine receptors on cells. Dopamine plays an important role in how we respond to “rewards”—things that make us feel good—and a reduction in dopamine receptors is associated with an increased risk of substance use disorder, commonly known as addiction.

How can prenatal THC exposure affect an individual’s behavior after birth and as they grow up? One way involves a biological process called epigenetics, in which chemical changes are made to the DNA, which can turn genes on or off. These changes are long-lasting but may be reversible. Studies have demonstrated that, if animals are exposed to THC during pregnancy, the infants after birth show changes in the on/off patterns of genes that regulate their behavior in adulthood [4]. These epigenetic changes help to explain why THC can cause such long-term effects on the brain. Also, since epigenetic mechanisms are reversible, this information gives us hope that we can develop treatments to reverse the effects of cannabis.

**Figure 1:**
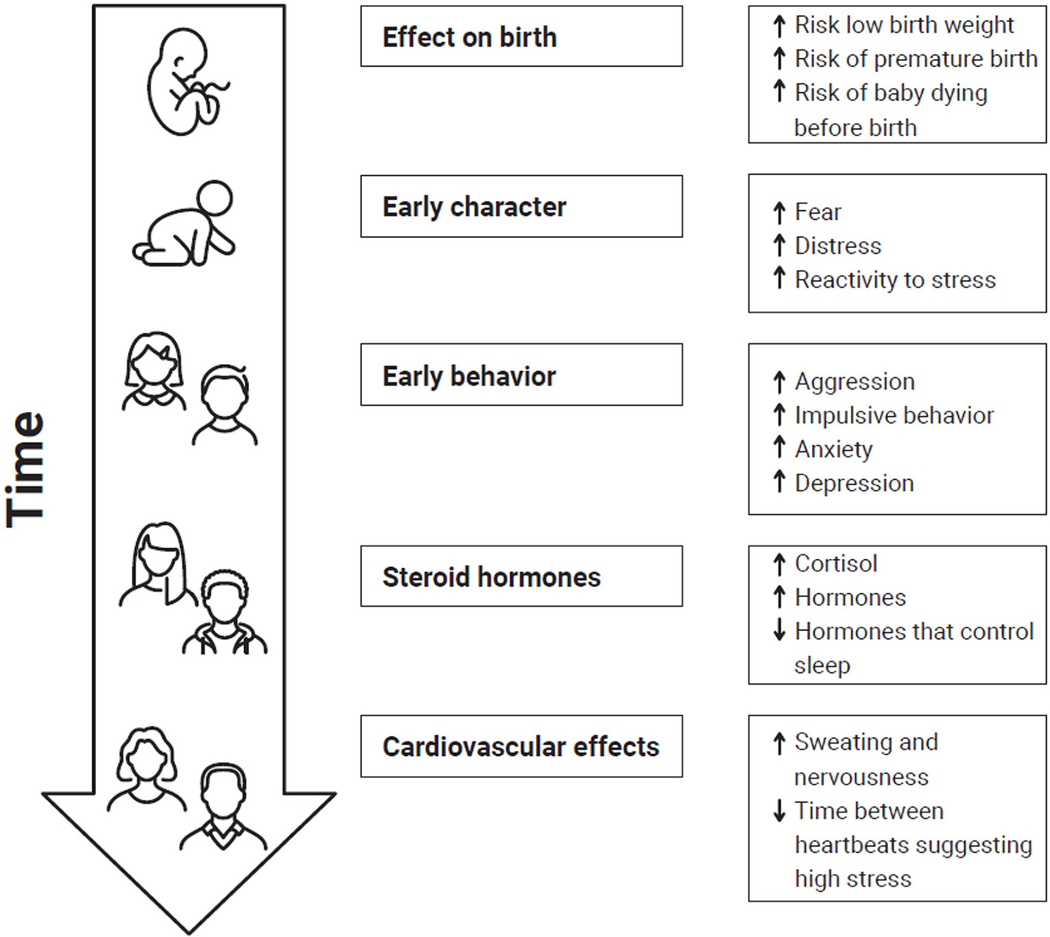
Exposure of unborn babies to THC or stress can have an impact on them for their whole lives, from before birth into adulthood. Response to stress and THC exposure to unborn babies is similar. This figures shows the effects of stress or THC exposure before birth at different stages of life (↑ = increase; ↓ = decrease).

Another study showed that exposure of babies to cannabis in the womb led to increased levels of the stress hormone cortisol in their bodies, increased anxiety, increased aggression, and an increase in disruptive behavior (Figure 1). Similar effects are seen in babies born to mothers who experience traumatic stress during pregnancy, such as coping with a natural disaster [3, 5]. This might mean that a mother who uses cannabis while pregnant might also be priming her baby’s brain to overreact when exposed to stress later in life [6].

## THC EXPOSURE DURING THE TEENAGE YEARS

There is a well-known link between cannabis use in teenage years and negative mental health outcomes such as mental illness, depression, anxiety, and addiction. Addiction to cannabis is called cannabis use disorder (CUD). CUD is on the rise, and risks for developing CUD peak during the teenage years. However, not everyone who tries cannabis will become addicted, and it is important to understand what makes some people more vulnerable to CUD. Studies have shown that teens and young adults who have both anxiety *and* genes that are related to addiction were much more likely to develop CUD than those with anxiety alone or addiction-related genes alone [7].

One of the strongest influences for developing CUD is the age at which cannabis is first used—the younger a person is when they first use cannabis, the more likely they are to develop CUD. For every year a teenager delays cannabis use, the risk of developing CUD drops by about 10%. Use of cannabis during the teen years is not just associated with an increased risk of CUD, but with an increased risk of addiction in general. So, delaying cannabis use until later in life may decrease some of the negative effects on the brain and behavior. Of course, if the use of cannabis is avoided altogether, the risks of CUD or other negative brain effects due to cannabis will be zero.

Since the concentration of THC in cannabis has more than quadrupled over time, teens today might be exposed to stronger cannabis than teens of the past, so understanding the effects of high doses of THC is crucial. Studies in animals have shown that high doses of THC activate the brain’s stress mechanisms more strongly than low-dose THC [8]. When these animals experienced a stressful event, such as being isolated from other animals, their reward-seeking behavior and social anxiety increased; and this was associated with changes in the structure of certain brain cells called astrocytes. Astrocytes support brain function by providing brain cells with nutrients, repairing them after injury, and helping them to communicate effectively. Changes to astrocytes caused by THC may contribute to increased anxiety, depression, and social avoidance [8].

## DECREASING THE RISKS

**Figure 2:**
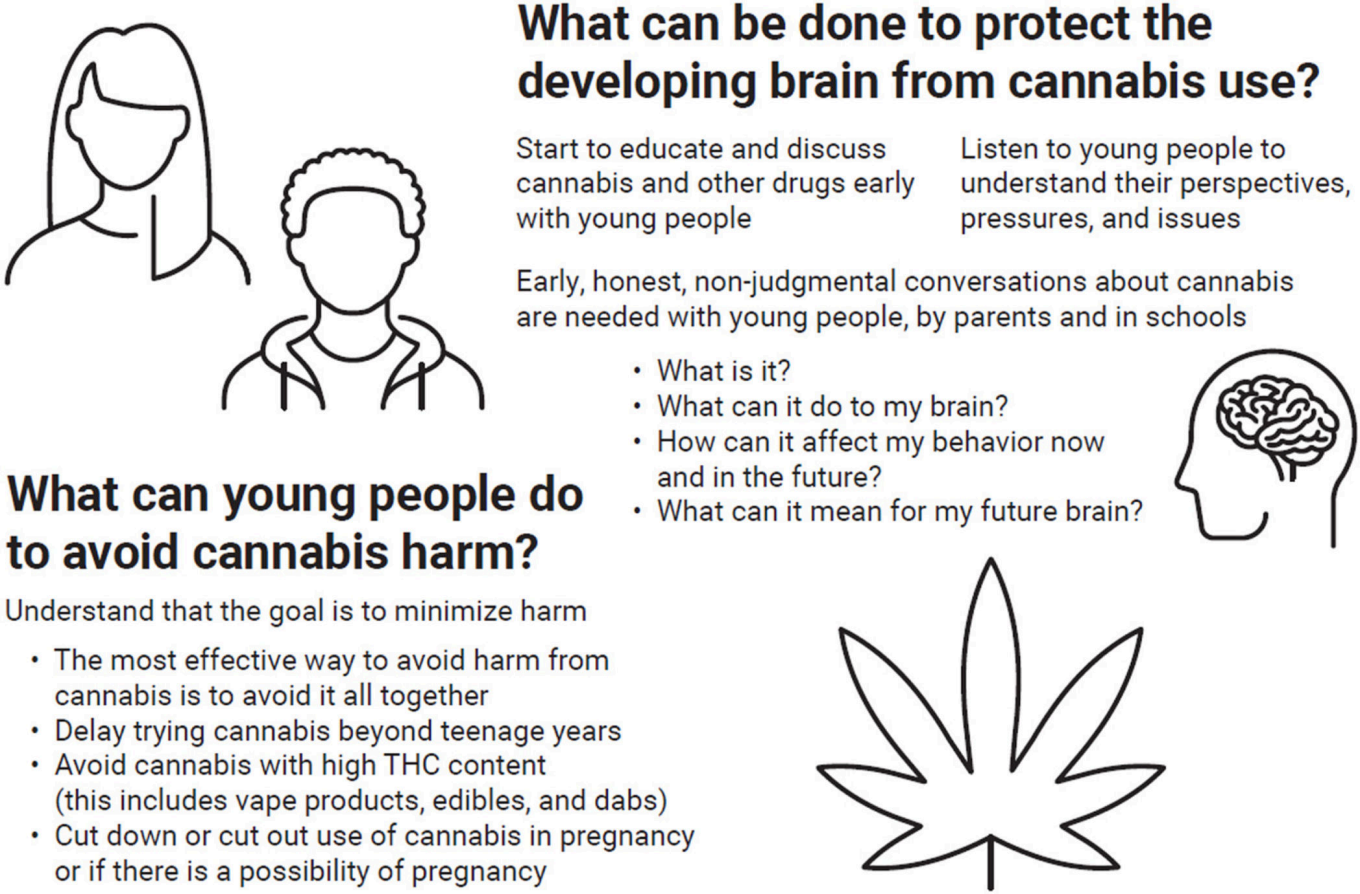
Since cannabis can affect the developing brain, it is critical for young people to minimize harm from cannabis use. This figure gives some ideas of how this might be achieved.

How can we minimize the risk of cannabis on the developing brain (Figure 2)? The best way to minimize risk is to avoid or reduce the use of cannabis! Learning the facts about the negative effects of cannabis on brain development is vital because this information can prevent people from starting to use cannabis in the first place. Trusted adults should start conversations about cannabis with young people early, ideally in the pre-teen years. Most often, we do not know whether we have the genes that contribute to addiction, or whether our mothers experienced stress or drug use during pregnancy—things that might influence our response to cannabis. As a result, we cannot know if exposing ourselves to cannabis in our teenage years will build on those unknowns and negatively impact our behavior, our mental health, or our future urge to use dangerous drugs.

Teenagers who are at high risk for cannabis use or are currently using cannabis in a problematic way should get treatment as soon as possible. Early treatment is most successful. If teenagers are unwilling to attend treatment, parents and caregivers should attend on their own—parental attendance has been shown to increase the willingness of young people to get treatment themselves.

## SUMMARY

**Figure 3:**
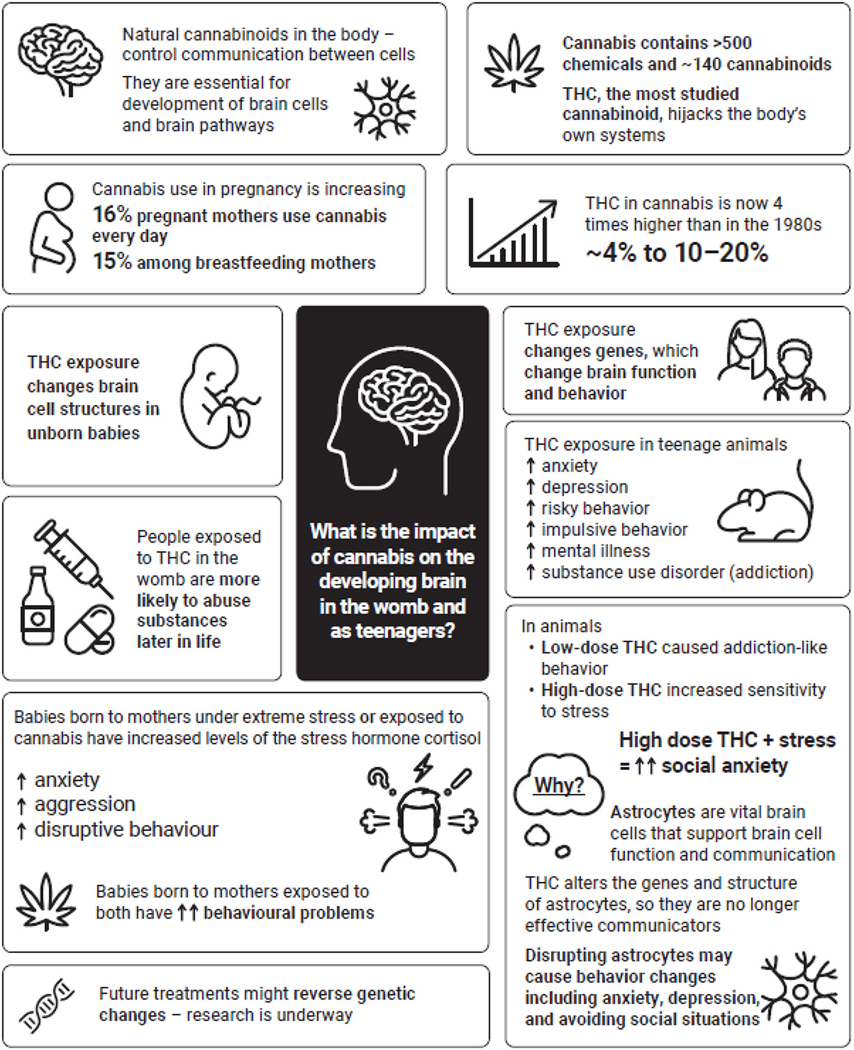
Summary of the effects of cannabis on the developing brain. (↑ = increase; ↑↑ = significant increase/greater-than-expected increase), THC, tetrahydrocannabinol.

Exposure to THC during important periods of brain development, particularly in the womb or during the teenage years, can cause long-lasting changes to the brains and behaviors of children—both in childhood and beyond (Figure 3). Avoiding the use of cannabis completely, or at least delaying its use until after the teenage years, is the best way to protect the brain. When we understand the effects of cannabis we can make informed choices—choices that can benefit our future brains and bodies!

## Abbreviations

CANNABINOIDS: The chemical compounds found in cannabis
ENDOCANNABINOIDS: Chemicals made within our own bodies that influence the brain and body in a very controlled way including emotion control, movement, memory
CANNABINOID RECEPTORS: Specific molecules which exist on the surfaces of many cell types which bind our natural endocannabinoids and cannabis
DOPAMINE: A body chemical that helps us feel good after “rewarding” behaviors
EPIGENETICS: A biological process by which a person’s environment or behavior impacts whether their genes are turned on or off
CORTISOL: A hormone released by the body in times of stress, it can increase anxiety, aggression, and disruptive behavior
CANNABIS USE DISORDER (CUD): Addiction to cannabis or problematic use of cannabis
ASTROCYTES: Star-shaped brain cells that perform a wide range of functions including providing nutrients to other brain cells and repairing them after injury
